# Bisphenol A and Metabolic Syndrome: Results from NHANES

**DOI:** 10.1155/2012/598180

**Published:** 2012-11-28

**Authors:** Srinivas Teppala, Suresh Madhavan, Anoop Shankar

**Affiliations:** ^1^Department of Epidemiology, West Virginia University School of Public Health, P.O. Box 9190, Morgantown, WV 26506-9190, USA; ^2^Department of Pharmaceutical Systems and Policy, West Virginia University School of Pharmacy, Morgantown, WV 26506, USA

## Abstract

*Background*. Bisphenol A (BPA) is detected in the urine of >95% of US adults. Recent evidence from population-based studies suggests that BPA is associated with individual components for metabolic syndrome (MetS). However, no previous study has examined the direct association between BPA and MetS. *Methods*. We examined 2,104 participants from the National Health and Nutrition Examination Survey 2003–2008. The main outcome was the presence of MetS (*n* = 741). *Results*. Increasing levels of urinary BPA were positively associated with MetS, independent of confounders such as age, gender, race/ethnicity, smoking, alcohol intake, physical activity, and urinary creatinine. Compared to tertile 1 (referent), the multivariable adjusted odds ratio (95% confidence interval) of MetS in tertile 3 was 1.51 (1.07–2.12); *P*-trend was 0.02. *Conclusions*. Urinary BPA levels are positively associated with MetS, in a representative sample of US adults and independent of traditional risk factors for MetS. Future, prospective studies are needed to confirm our findings.

## 1. Introduction

According to national biomonitoring surveys conducted by the Centers for Disease Control and Prevention, the plasticizer, bisphenol A (BPA), is present in the urine of more than 95% of US adults [1, 2]. BPA is an endocrine disruptor with estrogen-like effects. Studies based on animal models suggest that BPA exposure is associated with insulin resistance and weight gain. Previous studies including those from our group [3–5] suggest that higher BPA exposure is related to individual components of the metabolic syndrome (MetS) including hypertension [4, 6], diabetes mellitus [3, 7–9], insulin resistance [10], and obesity [5, 8, 11]. However, to our knowledge, there are no prior studies examining the association between BPA exposure and MetS in humans. In this context, we hypothesized that higher levels of BPA are independently associated to higher likelihood of MetS. We examined the association between urinary levels of BPA and MetS in the National Health and Nutrition Examination Survey 2003–2008 (NHANES), a large nationally representative multiethnic sample of US adults.

## 2. Methods

The current study is based on data from NHANES 2003–2008 [12–14]. In brief, the NHANES survey included a stratified multistage probability sample representative of the civilian noninstitutionalized US population. Subjects signed a consent form before their participation, and approval was obtained from the Human Subjects Committee in the U.S. Department of Health and Human Services [12–14].

The current study sample consisted of participants aged ≥18 years among whom urinary BPA was available (*N* = 5,231). We further excluded subjects with missing data on waist circumference, fasting serum glucose, serum triglycerides, serum high-density lipoprotein (HDL) for the definition of MetS, and other covariates used in our multivariable models. The final sample for the analysis comprised of 2,104 participants (51.0% women), of which 741 had MetS. Subjects who were excluded due to missing covariates were in general similar to the final study sample in terms of age, sex, race/ethnicity, smoking status, alcohol intake, and moderate physical activity (see the Appendix).

Rigorous procedures with quality control checks were used in blood and urine collection, and details about these procedures are available online [12–14]. Serum glucose, triglycerides, and HDL were measured enzymatically in the morning session (fasting sample) at the University of Missouri Diagnostic Laboratory. Seated systolic and diastolic blood pressures were measured using a mercury sphygmomanometer according to the American Heart Association and Seventh Joint National Committee (JNC7) recommendations [15]. Up to 3 measurements were averaged for systolic and diastolic pressures. Urinary creatinine analysis was based on the Jaffe reaction and using CX3 analyzer.

Previous measures of BPA in biological matrixes involved techniques such as gas chromatography or high performance liquid chromatography [16]. To achieve enhanced sensitivity and selectivity over previous methods, in the current NHANES, measures of environmental phenols were derivatized to alkyl or acyl derivatives before gas chromatography/mass spectrometry analysis [17–19]. The lower limit of detection for BPA concentrations was 0.36 ng/mL.

MetS was defined based on the revised Adult Treatment Panel III (ATP III) guidelines [20] and included the following criteria: (1) abdominal obesity (waist circumference: ≥102 cm in men and ≥88 cm in women), (2) hypertension (systolic blood pressure ≥130 mm of Hg, diastolic blood pressure ≥85 mm of Hg, use of medications for elevated blood pressure), (3) elevated serum triglycerides (≥150 mg/dL), (4) glucose intolerance (fasting serum glucose ≥100 mg/dL, medications for diabetes), and (5) reduced HDL (<40 mg/dL for men and <50 mg/dL for women). Participants positive for 3 or more of the 5 measured components were considered to have MetS.

## 3. Statistical Analysis

Urinary BPA was categorized into tertiles (<1.4 ng/mL, 1.4–3.4 ng/mL, and >3.4 ng/mL). We hypothesized that high BPA levels are associated with MetS. The odds ratio [(OR) (95% confidence interval (CI)] of MetS for BPA was calculated by taking the lowest tertile (tertile1) as the referent, using multivariable logistic regression models. We used two models: the age and sex-adjusted model and the multivariable model, additionally adjusting for race/ethnicity, annual household income, smoking status, alcohol intake, moderate physical activity, and urinary creatinine (mg/dL). Trends in the OR of MetS across increasing urinary BPA categories were determined by modeling BPA as an ordinal variable. Sample weights that account for the unequal probabilities of selection, oversampling, and nonresponse were applied for all analyses using SAS (version 9.3; SAS Institute, Cary, NC, USA) and SUDAAN software; standard errors (SEs) were estimated using the Taylor series linearization method.

## 4. Results

Table 1 shows the characteristics of the whole cohort. Participants with higher levels of BPA were more likely to be of non-Hispanic White race/ethnicity, currently smoking, have higher levels of alcohol intake, higher triglycerides, lower HDL, and higher urinary creatinine levels.

In Table 2, we examine the association between BPA and MetS. Overall, increasing levels of BPA were associated with MetS in both the age and sex-adjusted model and the multivariable model, where we additionally adjusted for race/ethnicity, smoking status, alcohol intake, annual household income, moderate physical activity, and urinary creatinine levels; *P* values for trend were also significant (*P*-trend = 0.02). 

## 5. Discussion

In a representative sample of US adults, we found that increasing levels of urinary BPA were positively associated with MetS. The observed association was found to be independent of confounding factors such as age, gender, race/ethnicity, smoking, alcohol intake, moderate physical activity levels, and urinary creatinine. To our knowledge, our study is the first report on a positive association between BPA and MetS in humans.

BPA is a constituent monomer in polycarbonate plastics, used extensively in the manufacture of several consumer grade products including food packaging, bottled water and the lining of canned foods [21]. Recent studies have documented that over 95% of the US population have measurable concentrations of BPA in their urine [1, 2].

Several lines of recent evidence suggest that our findings of an association between BPA and MS are plausible. Previous studies based on animal models provide evidence towards both the endocrine disrupting and estrogen-like effects of BPA and suggest that BPA exposure is related to increased insulin resistance [22] and, therefore, has a role in weight gain and the development of obesity [5, 8, 23]. In an experimental study on human tissue explants, Hugo et al. found that increasing levels of BPA inhibit the release of the beneficial lipid modulating hormone adiponectin, implicating BPA in the development of insulin resistance and MetS [24]. Furthermore, BPA was also associated with known MetS risk factors such as hypertension [4, 6] and diabetes [3, 7–9] in previous studies.

The main strengths of our study include its nationally representative sample, use of rigorous study methods to collect the data, and the availability of extensive data on confounders [12–14]. The main study limitation is that the current study is cross-sectional in nature, therefore, making it impossible to draw cause effects in the observed associations. Another concern to our observed association between BPA levels and MetS is the potential confounding role of diet. It has been shown that majority of BPA exposure in humans is dietary (e.g., the modern Western fast-food diet and drinking canned/bottled sodas) [25], and the same types of diet have been shown to be associated with MetS [26]. Also, recent reports on the pharmacokinetics of BPA suggest that in humans most of BPA is metabolized before it reaches the systemic circulation [27, 28]; therefore, the observed BPA-MetS association may be noncausal and confounded by unhealthy diet. 

Previous studies have suggested the inclusion of several additional markers associated with insulin resistance and increased cardiovascular risk, such as hyperuricemia [29], nonalcoholic fatty liver disease [30], polycystic ovary syndrome [31], and hypogonadism [32] towards the assessment of MetS. However, in the current study, our definition of MetS is based on the revised ATP III guidelines [20], which do not include these markers. Since BPA may be positively associated with many of these markers, using the ATP III definition for MetS may have attenuated our observed association. Future studies on BPA and MetS should try to also incorporate these newly suggested markers of MetS to study the true association.

In conclusion, we found that in a nationally representative sample of US adults, higher exposure to BPA was positively associated with MetS independent of confounding factors such as age, gender, race/ethnicity, smoking, alcohol, moderate physical activity and urinary creatinine. If confirmed in future prospective studies, reducing environmental exposure to BPA may potentially have a role in the prevention of MetS.

## Figures and Tables

**Table 1:** Characteristics of the study population from NHANES 2003–2008^a^.

Characteristics	Tertile 1 (*N* = 695)	Tertile 2 (*N* = 704)	Tertile 3 (*N* = 705)	*P* value
Age (years)	48.29 ± 0.74	45.24 ± 0.73	42.26 ± 0.64	<0.0001
Female (%)	381 (56.3)	338 (48.4)	343 (48.4)	0.03
Race/ethnicity (%)				
Non-Hispanic Whites	367 (75.3)	357 (71.8)	314 (67.5)	0.02
Non-Whites	328 (24.7)	347 (28.2)	391 (32.4)	
Smoking status (%)				
Never smoker	351 (50.8)	338 (49.9)	309 (44.5)	0.05
Former smoker	186 (26.8)	177 (24.5)	147 (23.8)	
Current smoker	121 (20.2)	141 (22.5)	161 (26.4)	
Missing	37 (2.3)	48 (3.1)	88 (5.2)	
Alcohol intake (%)				
Nondrinker	291 (31.8)	272 (33.1)	312 (35.4)	<0.0001
Moderate drinker	278 (49.7)	249 (39.7)	221 (36.2)	
Heavy drinker	126 (18.4)	183 (27.2)	172 (28.4)	
Annual household income				
<$20,000	129 (10.8)	133 (12.7)	160 (15.7)	0.02
$20,000–$54,999	261 (32.4)	269 (35.9)	287 (38.8)	
≥55,000	257 (51.8)	250 (45.2)	224 (40.9)	
Missing	48 (4.9)	52 (6.2)	34 (4.6)	
Moderate physical activity (%)	233 (42.4)	251 (40.1)	265 (39.9)	0.69
Waist circumference (cm)	95.97 ± 0.74	97.81 ± 0.85	97.86 ± 0.89	<0.0001
Systolic blood pressure (mm of Hg)	120.56 ± 0.76	120.76 ± 0.60	119.58 ± 0.81	<0.0001
Diastolic blood pressure (mm of Hg)	70.17 ± 0.66	69.65 ± 0.57	69.48 ± 0.54	<0.0001
Serum triglycerides (mg/dL)	129.61 ± 3.84	138.16 ± 5.88	145.81 ± 5.32	<0.0001
Serum HDL (mg/dL)	57.19 ± 0.86	54.86 ± 0.81	52.20 ± 0.65	<0.0001
Serum glucose (mg/dL)	102.78 ± 1.22	102.64 ± 1.02	104.85 ± 1.58	<0.0001
Urinary creatinine (mg/dL)	85.03 ± 2.57	132.83 ± 3.07	175.80 ± 4.14	<0.0001
Metabolic Syndrome (%)	30.0	34.9	38.9	0.01

^a^Data presented are number (percentages) or mean values ± standard error (SE), as appropriate for the variable.

**Table 2:** Association between bisphenol A and metabolic syndrome.

Bisphenol A (ng/mL)	Sample size	Age, sex-adjusted OR	Multivariable-adjusted OR
	(% MetS)	(95% CI)^a^	(95% CI)^a,b^
Tertile 1 (<1.40)	695 (30.0)	1 (referent)	1 (referent)
Tertile 2 (1.40–3.40)	704 (34.9)	1.26 (0.95–1.68)	1.24 (0.93–1.65)
Tertile 3 (>3.40)	705 (38.9)	1.53 (1.09–2.15)	1.51 (1.07–2.12)
*P*-trend		0.01	0.02

^a^OR (95% CI): odds ratio (95% confidence interval).

^b^Adjusted for age (years), gender (male, female), race/ethnicity (non-Hispanic Whites, non-Hispanic Blacks, Mexican Americans, and others), annual household income, smoking (never, former, and current), alcohol intake (nondrinker, moderate drinker, and heavy drinker), moderate physical inactivity (absent, present), and urinary creatinine (mg/dL).

**Table 3:** Comparison of those who were included the final analytic sample to those who were excluded due to missing data^a^.

Characteristics	Final analytic sample (*N* = 2,104)	Missing data (*N* = 3,127)	*P* value
Age (years)	45.3 ± 0.5	45.4 ± 0.4	<0.0001
Female (%)	1,062 (51.0)	1,625 (52.1)	0.54
Race/ethnicity (%)			0.29
Non-Hispanic Whites	1,038 (71.6)	1,486 (70.0)	
Non-Whites	1,066 (28.4)	1,641 (30.0)	
Smoking status (%)			0.64
Never smoker	998 (48.4)	1,497 (49.9)	
Former smoker	510 (25.0)	713 (23.0)	
Current smoker	423 (23.0)	645 (23.3)	
Missing	173 (3.5)	271 (3.7)	
Alcohol intake (%)			0.10
Nondrinker	875 (33.4)	1,448 (36.4)	
Moderate drinker	748 (41.9)	1,022 (39.0)	
Heavy drinker	481 (24.6)	657 (24.6)	
Annual household income			0.02
<$20,000	422 (13.0)	733 (15.9)	
$20,000–$54,999	817 (35.6)	1,215 (35.7)	
≥55,000	731 (46.0)	942 (42.0)	
Missing	134 (5.2)	237 (6.3)	
Moderate physical activity (%)	749 (40.8)	1,075 (38.3)	0.18

^a^Data presented are number (percentages) or mean values ± standard error (SE), as appropriate for the variable.

## Appendix

See Table 3.
